# IgG4-related Breast Disease: Review of the Literature

**DOI:** 10.5041/RMMJ.10532

**Published:** 2024-10-28

**Authors:** Helana Jeries, Yolanda Braun-Moscovici, Alexandra Balbir-Gurman

**Affiliations:** 1 Rheumatology Unit, Galilee Medical Center, Nahariya, Israel; 2 The Azrieli Faculty of Medicine, Bar-Ilan University, Safed, Israel; 3 The B. Shine Rheumatology Institute, Rambam Health Care Campus, Haifa, Israel; 4 Ruth and Bruce Rappaport Faculty of Medicine, Technion–Israel Institute of Technology, Haifa, Israel

**Keywords:** IgG4-related disease, IgG4-related mastitis, IgG4-related breast lump

## Abstract

IgG4-related disease (IgG4-RD) is a rare illness with inflammatory and fibrotic changes in affected organs such as pancreas, thyroid, salivary or lacrimal glands, and retroperitoneal space; rarely other organs may be involved. IgG4-related breast disease (IgG4-BD) is very rare and generally presents as a lump or mastitis. IgG4-BD as a presenting feature of IgG4-RD is extremely rare. Hence, this paper reviews the known (*n*=48) IgG-BD cases reported in the literature to date. The majority of cases were diagnosed on routine mammography or during assessment for other clinically significant features. The absence of a lump border, and especially the absence of calcifications on ultrasonography, mammography, or computed tomography, is typical for IgG4-BD. Characteristic IgG4-BD pathological findings were dense lymphoplasmacytic infiltration with stromal fibrosis, and more than 10% IgG4^+^ plasma cells/high-power field (HPF); the mean percentage of IgG4^+^/IgG^+^ plasma cells was 54.2%, and only one-third of the patients had all “classical” signs of IgG4-BD including storiform fibrosis and obliterative phlebitis. Most of the cases had a benign course and responded to surgical excision with or without steroid therapy.

## INTRODUCTION

IgG4-related disease (IgG4-RD) was defined in 2003 by Kamisawa et al., who described it as an immune-mediated fibro-inflammatory disorder that can affect heterogeneous organs (seen as pancreatitis, sialadenitis, retroperitoneal fibrosis, and thyroid and lacrimal glands involvement), with particular attention to pancreatitis.^1^ Clinical presentation of IgG4-RD may include systemic signs, such as fatigue and weight loss, in combination with organ-specific symptoms. The latter may include salivary gland enlargement, abdominal pain, and/or jaundice in cases of pancreatic or biliary involvement, hydronephrosis and renal failure in cases of retroperitoneal fibrosis, pericardial effusion and/or thickening, peri-aortitis, and periorbital mass.^2^ However, IgG4-RD presented with breast mass or mastitis is uncommon. Moreover, IgG4-related breast disease (IgG4-BD) concomitant with “classical” IgG4-RD target organ involvement is very rare. IgG4-BD generally presents as an asymptomatic unilateral inflammatory tumor-like mass or mastitis with or without inflammatory skin infiltration; in some patients, nipple retraction and regional axillary lymphadenopathy have been reported.^3^

The etiology of IgG4-RD in general and IgG4-BD in particular is unknown. Biopsies of affected tissues demonstrated infiltration by lymphocytes, mast cells, macrophages, and fibroblasts, accompanied with overproduction of profibrotic factors such as CCL-18, interleukin (IL)-33, IL-4, IL-13, IL-1β, tumor growth factor-β, and interferon-γ.^4^

The true prevalence and incidence of IgG4-RD as well as IgG4-BD are undetermined.^2^,^5^,^6^ Inoue et al. reported a dataset of 235 patients with IgG4-RD: 91% were over 50 years of age; 80.2% were males; 42% had isolated lesions, while the rest had multiple organ involvement; 19 patients (8.1%) had previous autoimmune disease, and 1 patient (0.4%) had rheumatoid arthritis.^5^ Wallace et al. reported a series of 125 patients with IgG4-RD with a mean age of 55.2 (24–83) years and male predominance (61%).^6^ There were no cases of IgG4-BD in any of these cohorts, confirming further how rare IgG4-BD is.^5^–^7^

Typically, IgG4-RD is characterized by the appearance of fibro-inflammatory lymphocytic infiltrates in affected organs causing a variety of clinical presentations such as organ inflammation, dysfunction, obstruction, or tumor-like lesions. Imaging, including ultrasonography (US), computed tomography (CT), and magnetic resonance imaging (MRI), of involved organs is very helpful for demonstrating structural changes in the involved organ but is not specific. Elevated serum IgG4 levels occur in 50%–90% of patients, depending on the clinical phenotype and affected organ; serum levels of IgG4 were validated and defined as clinically significant if they exceeded 135 mg/dL.^6^,^8^,^9^ In cases with prominent systemic features such as prolonged fever, and/or weight loss, and/or lymphadenopathy, assessment with ^18^F-fluorodeoxyglucose (^18^F-FDG) positron emission tomography–CT (PET-CT) is helpful.^10^

Biopsies from involved tissue generally demonstrate characteristic histopathologic findings such as dense polyclonal lymphoplasmacytic and mild eosinophilic infiltrates, high percentages of IgG4^+^ plasma cells (>10%/HPF), IgG4^+^ cells/IgG^+^ cells percentage greater than 40%, storiform fibrosis, and obliterative phlebitis.^11^ In contrast to the “classic” histological patterns of IgG4-RD organ involvement, in IgG4-BD the “full house” combination of these pathological features is rare; IgG4-BD biopsies mainly showed lymphoplasmacytic infiltrates with glandular atrophy, while storiform fibrosis and obliterative phlebitis were only rarely described.^12^

The American College of Rheumatology (ACR)/European Alliance of Associations for Rheumatology (EULAR) IgG4-RD classification criteria (of 2019) proposed a positive diagnosis of IgG4-RD in cases meeting the entry criteria in the absence of exclusion criteria and achieving a total point score of ≥20 in eight different domains.^13^ Interestingly, in the three large derivation and validation cohorts that were analyzed (486, 908, and 485 patients) as part of their project to determine classification criteria, there were no cases of IgG4-BD. This, again, particularly calls out the rarity of breast involvement in IgG4-RD.

## IgG4-RELATED BREAST DISEASE LITERATURE REVIEW

A literature search was performed in PubMed, MEDLINE, and Google Scholar for articles about IgG4 breast disease published in English between January 1, 1980 and September 1, 2023. The keywords used were: “IgG4 related breast disease,” “IgG4 related disease,” “breast,” “involvement,” and “IgG4.” A total of 25 reports on 48 cases had been published in the English language literature^14^–^38^ (see Table 1, provided as a supplement for easier reading). Some reports included full clinical and histological aspects of described cases, while others provided only limited clinical information and were primarily based on retrospective studies of biopsied samples. Of the 48 published cases, 46 were female (96%) with a mean age of 56.02 years (standard deviation [SD] 13.43). Only 6 patients had painful lesions (12.5%). Breast lumps were generally discovered during routine clinical or mammography (MG) assessment or while evaluated for another suspected pathology. In 28 cases information was available regarding lump location: 27 (96.42%) patients had unilateral disease (16, right; 10, left; 1, side not reported), and 1 patient had bilateral lesions. We found 18 patients with data on axillar status, 5 (27.7%) of whom had lymphadenopathy. A few patients had information on additional IgG4-RD organ involvement: 6 in the lacrimal glands, 3 in the pancreas, 2 of the skin, 2 with salivary glands, and 1 each in the lung nodes, lymph nodes, and retroperitoneum; 5 patients had more than two organs involved. Of the 14 cases with reference to systemic signs, only 1 patient had fever; data on CRP were available in only 2 patients, elevated in both cases. Of the 22 cases for which comorbidity reports were available, 11 patients had previous diseases: 3, thyroid; 4, hypertension; 2, lung disorders (1 with chronic obstructive pulmonary disease; 1 with interstitial lung disease); 1, Sjogren’s disease and Hodgkin lymphoma; and 1, prostatic cancer.

A breast lump is highly suspicious for malignancy and is usually assessed with MG, US, and/or MRI. On mammography IgG4-BD is usually seen as a mass without clear borders and generally without pathologic calcifications; the same is true for CT breast assessment. On US, IgG4-BD lesions appear as an ill-defined hypoechoic mass with low or absent vascularity, no acoustic shadowing, and no calcifications. On MRI, the lump generally presents as non-mass lesion with skin enhancement, and on PET-FDG, an IgG4-related lesion has high fluorodeoxyglucose uptake. In this review, 40 patients underwent MG, 22 US, 3 chest CT, 4 PET-FDG, and 7 MRI.

All 48 had breast biopsy to rule out malignancy, 2 of whom additionally underwent lacrimal gland biopsy. All but one biopsied sample was negative for breast cancer; in one case there was concomitant IgG4-BD and *de novo* breast carcinoma.^28^

Data on serum IgG4 levels were provided for 39 patients, with a mean level of 404.23 ng/dL (SD 834.5). The histopathology and immunohistochemistry staining observed in IgG4-BD were quite diverse in the presented reports: the majority of lesions were characterized by a robust presence of lymphocytes and plasma cells, with more than 10% IgG4^+^ plasma cells per HPF, and an elevated IgG4^+^/IgG^+^ plasma cell percentage. In a few lesions, eosinophils and/or histiocytes were observed. In our analysis, only 16 patients (31.4%) exhibited all “classical” signs of IgG4-BD, including storiform fibrosis and obliterative phlebitis. Descriptions of dense lymphoplasmacytic infiltrate with stromal fibrosis and more than 10% IgG4^+^ plasma cells per HPF were noted in 46 patients. The mean percentage of IgG4^+^/IgG^+^ plasma cells was 54.2% (data on 38 patients; SD 16.37). Three patients had an overlap between IgG4-BD and Rosai–Dorfman disease.^32^,^35^ As already mentioned, IgG4-BD was occasionally identified in samples that tested negative for breast cancer, following additional staining for IgG4. Among 63 breast biopsies with initial IgG4 staining, 17 cases of granulomatous mastitis were found. Of these, 5 (29%) showed IgG4 positivity with 5%–10% IgG4^+^ plasma cells. In all 17 samples, the IgG4/IgG percentage was >35%.^39^

Guidelines for assessment, diagnosis, and treatment of IgG4-RD were recently published.^40^ They particularly pointed out the destructive nature of lesions and recommended treatment for both symptomatic *and* asymptomatic patients, to prevent irreversible tissue and organ damage. Corticosteroids are the first line of treatment, particularly in patients with “urgent” disease (pancreatic, aortic, meningeal, or pericardial involvement). The use of steroid-sparing agents is questionable. However, the use of rituximab, a monoclonal antibody, seems promising for induction and maintenance therapy, especially in light of the high incidence of IgG4-RD relapses.^40^ Regarding the treatment of analyzed cases in this review, 2 patients with clinical mastitis and fluid collection observed by US were treated with drainage; 3 patients received antibiotics without any effect. Information on treatment modalities was available for only 24 patients in this review: 2 patients were managed with observation only; 13 underwent surgical lump excision, and 9 were treated with steroids, mainly at an initial dose of 30 mg/day. Among the 17 patients with known outcomes, the course of IgG4-BD was generally benign.

## CONCLUSION

The key findings of this study are outlined in Box 1; IgG4-RD can affect nearly any organ, and IgG4-BD is very uncommon, particularly as an initial presentation. The majority of cases are diagnosed on routine MG or during assessment for other clinically significant features. Lump border absence is typical for IgG4-BD, as is especially the absence of calcifications on US, MG, or CT. Concomitant involvement of other specific IgG4-RD tissues and elevated serum IgG4 levels may be a clue to consider IgG4-RD in the diagnostic process. Typical findings on biopsy together with special IgG4 staining are a gold standard for IgG4-RD diagnosis in many tissues; but the pathological criteria for IgG4-BD should be less strict since, in many cases, storiform fibrosis and obliterative phlebitis are absent. IgG4-BD responds well to steroids with low relapse tendency. Clinicians should be aware that IgG4-BD is probably more common than might be supposed, especially in so-called “negative for cancer” breast biopsies; hence, the awareness of IgG4-BD should be increased.

Box 1

***Take-home Messages***
1. IgG4-BD is uncommon, yet important to consider in patients with biopsies negative for malignancy.
2. The absence of lump border, and especially the absence of calcifications on US, MG, or CT, is typical for IgG4-BD.
3. Typical pathological findings of IgG4-BD include dense lymphoplasmacytic infiltration with more than 10% IgG4^+^ plasma cells/HPF and a percentage of IgG4^+^/IgG^+^ plasma cells >40%, while storiform fibrosis and obliterative phlebitis are absent in most cases.

## Supplementary Information



## Abbreviations

^18^F-FDG: ^18^F-fluorodeoxyglucose
ACR: American College of Rheumatology
CT: computed tomography
EULAR: European Alliance of Associations for Rheumatology
HPF: high-power field
IgG4-BD: IgG4-related breast disease
IgG4-RD: IgG4-related disease
IL: interleukin
MRI: magnetic resonance imaging
PET-CT: positron emission tomography/computed tomography
US: ultrasonography
