# Supplementary material for: IgG4-related Breast Disease: Review of the Literature
Source: Rambam Maimonides Med J. 2024 Oct 28;15(4):e0018. doi: 10.5041/RMMJ.10532 (PMC11524419; doi:10.5041/RMMJ.10532)
Supplement: Supplementary file 1 [file rmmj-15-4-e0018AM.pdf]

SUPPLEMENTARY MATERIAL

This appendix has been provided by the authors for the benefit of readers

Supplement to IgG4-related Breast Disease: Review of the Literature

Jeries H, Braun-Moscovici Y, Balbir-Gurman A. IgG4-related Breast Disease: Review of the Literature. Rambam Maimonides Med J 2024;15 (4):e0018. Review. doi:10.5041/RMMJ.10532

Table 1. Clinical, Imaging, and Pathological Data of IgG4-BD Cases Reported in the Literature.

Pt. # (age, y/sex)[ref.]	Presentation	Imaging	Biopsy	Serum IgG4 (mg/dL)	Histopathology	% $IgG4^+/IgG^+$	Treatment	Outcome
Pt. 1 (24/F)[14]	Pain and redness in right breast	**US:** Breast abscess	Vacuum and excisional	142	Focal architectural distortion, dense inflammatory infiltrate with dense sclerotic stroma. No granulomas	45	Prednisone 1 mg/kg<br>MTX 15 mg/week	Resolution within 3 months
Pt. 2 (70/F)[15]	Axillary lymph nodes, soft egg-sized tender mass in left breast	**MG:** Breast lump<br>**US:** Hypoechoic mass with irregular border and poor Doppler signal<br>**MRI:** Breast mass—suspected tumor	Yes, type not defined	—	Mixed T and B lymphocytic and plasma cell infiltration with interstitial fibrosis	40	Prednisone 10 mg/day	Lesion resolution stable during 4-year follow-up
Pt. 3 (56/F)[16]	Palpable non-painful lump in left breast	**US:** Ill-defined hypoechoic mass with low vascularity; no shadowing or calcification<br>**MRI:** Non-mass enhancement	Yes, type not defined	—	IgG4-related sclerosing mastitis	—	—	—

Pt. # (age, y/sex)ref.	Presentation	Imaging	Biopsy	Serum IgG4 (mg/dL)	Histopathology	% $IgG4^+/IgG^+$	Treatment	Outcome
Pt. 4 (52/F)[17]	Asymptomatic left breast lump om screening mammography	**MG:** Small circumscribed opacity<br>**US:** Small solid avascular hypoechoic nodule with hyperechogenic tiny foci	Core	131	Scattered stromal cells with chronic dense inflammatory infiltrates and prominent IgG4 expression	—	Excision	—
Pt. 5 (71/F)[18]	Asymptomatic left breast mass on CT (screening for IgG4-RD because of eye involvement)	**MG and US:** Breast mass—suspected tumor<br>**MRI:** Bordered breast mass with early enhancement—suspected tumor	Excision of large nodules	1430	Dense lymphoplasmacytic infiltration with $CD20^+$ B cells and $CD3^+$ T cells	76	Prednisone 22.5 mg/day, tapered to 5 mg/day as maintenance	Improved within 3 months; stabilized after 1 year
Pt. 6 (84/F)[19]	Palpable unilateral non-painful lump (unknown side)	**MG:** Breast mass without calcifications or architectural distortion<br>**US:** Well circumscribed round hypoechoic mass with vascular flow on Doppler	Excision of large nodules	—	Fibro-inflammatory lesion with dense lymphoplasmacytic infiltration, numerous plasma cells, eosinophils	50	Mass excision	—
Pt. 7 (56/F)[20]	Palpable bilateral non-painful lump	**MG:** Mass-like densities, both breasts<br>**US:** Irregular ill-defined hypoechoic masses<br>**PET:** Mildly increased metabolic activity	Core biopsy	Elevated	Extensive stromal fibrosis with robust $IgG4^+$ plasma cells in breast and dermal tissue	40	Steroids	Orbital disease resolution; stable breast mass
Pt. 8 (56/F)[21]	Recurrent mastitis (unknown side)	—	Excision of large nodules	920	IgG4 infiltrates	—	Prednisone 40 mg/day tapered to 10 mg/day maintenance therapy	Symptom resolution; stable after 1 year
Pt. 9 (41/F)[22]	Asymptomatic right breast lump on screening mammography	**MG and US:** Small mass	Biopsy	39.4	Sclerotic stroma with dense lymphoplasmacytic infiltrates	43	Observation	—
Pt. 10 (54/F)[23]	Palpable non-painful left breast lump	**US:** Irregular ill-defined hypoechoic masses<br>**CT:** Axillary nodes enlargement	Biopsy	3320	Mixed B, T, and plasma cell infiltrates with interstitial fibrosis	40	Prednisone 30 mg/day for 4 weeks and maintenance therapy	Decrease in breast mass

Pt. # (age, y/sex)ref.	Presentation	Imaging	Biopsy	Serum IgG4 (mg/dL)	Histopathology	% $IgG4^+/IgG^+$	Treatment	Outcome
Pt. 11 (48/F)[24]	Palpable bilateral non-painful lump	**MG:** Breast nodules<br>**US:** Hypoechoic nodules with lobular margins<br>**MRI:** Multiple lesions, both breasts	US-guided biopsy	650	Dense mass, lymphoplasmacytic infiltrates, prominent stromal sclerosis, loss of breast lobules; mostly B-$CD20^+$ and T $CD3^+$ lymphocytes	65	Excision of large nodules	Stable state
Pt. 12 (51/F)[24]	Palpable right breast non-painful lump	**MG:** Breast nodules	Core biopsy	3900	Dense mass, lymphoplasmacytic infiltrates, prominent stromal sclerosis, loss of breast lobules; mostly B-$CD20^+$ and T $CD3^+$ lymphocytes	85	Excision of large nodules	Stable state
Pt. 13 (37/F)[24]	Palpable right breast non-painful lump	**MG:** Breast nodules	Core biopsy	—	Dense mass, lymphoplasmacytic infiltrates, prominent stromal sclerosis, loss of breast lobules; mostly B-$CD20^+$ and T $CD3^+$ lymphocytes. Phlebitis	82	Core needle biopsy	Breast mass resolved, lymphadenopathy unchanged
Pt. 14 (54/F)[24]	Palpable right breast non-painful lump	**MG and US:** Fibro-adenoma-like lesions	Performed	—	Dense mass, lymphoplasmacytic infiltrates, prominent stromal sclerosis, loss of breast lobules; mostly B-$CD20^+$ and T $CD3^+$ lymphocytes	49	Excision of large nodules	Stable state
Pt. 15 (62/F)[25]	Painful right breast mass	**US:** Hypoechoic mass	US-guided	—	Granulomatous lobular mastitis with giant cells, sclerosis, and $IgG4^+$	—	—	—
Pt. 16 (66/F)[25]	Painful right breast mass	**US:** Ill-defined hypoechoic mass	Surgical excision	189	—	—	—	—
Pt. 17 (49/F)[26]	Multiple painless masses in left breast	**US:** Hypoechoic uncompressible mass, surrounding architecture disturbance without acoustic shadowing	Biopsy excision	390	Sclerotic lesions with low cellularity and plasma cell-rich inflammatory infiltrate	51	Steroids	Partial regression
Pt. 18 (47/F)[27]	Asymptomatic breast lump (unknown side)	—	—	140	Reactive lymphoid follicles, fibrosis, elevated eosinophils and plasma cells, no phlebitis	30	—	Recurrent orbital mass
Pt. 19 (61/F)[28]	Asymptomatic right breast lesion on planned PET-FDG	**MG and US:** Performed, no description<br>**PET-CT:** Increased meta-bolic activity in the breast (planned exam for lymphoma)	—	321	Sclerotic lesions with low cellularity and plasma cell-rich inflammatory infiltrate: lymphocytes, plasma cells, eosinophils, histiocytes. Carcinoma *in situ*	85	Excision	—

Pt. # (age, y/sex)[ref.]	Presentation	Imaging	Biopsy	Serum IgG4 (mg/dL)	Histopathology	% $IgG4^+/IgG^+$	Treatment	Outcome
Pt. 20 (59/M)[28]	Asymptomatic left breast lesion on planned total body CT	**MG:** Multinodular irregular mass<br>**CT:** For prostatic cancer assessment, breast mass	—	—	Stromal sclerosis with dense plasma cells (>30 HPF) infiltrate, positive for IgG4	—	Excision	—
Pt. 21 (48/M)[29]	Asymptomatic right breast lump	**MG:** Breast mass involving pectoral muscle with skin thickening<br>**US:** Heterogeneous mass with mild vascularity	—	1220	Fibrotic tissue with storiform pattern and dense lympho-plasmacytic infiltrates, several eosinophils, no granuloma	51	Excision	—
Pt. 22 (66/F)[30]	Asymptomatic left breast lump	**MG:** Breast mass—suspected tumor	—	179	Storiform fibrosis, dense lymphoplasmacytic infiltrates, obliterative phlebitis	63.9	Excision	—
Pt. 23 (45/F)[30]	Asymptomatic right breast lump	**MG:** Breast mass—suspected tumor	—	308	Storiform fibrosis, dense lymphoplasmacytic infiltrates, obliterative phlebitis	67.3	Excision	—
Pt. 24 (37/F)[31]	Asymptomatic breast lump (unknown side)	**MG:** Breast lesion	Performed	166	Dense lymphoplasmacytic infiltrates, storiform fibrosis, obliterative phlebitis, giant cells	75.1	—	—
Pt. 25 (—/—)[31]	Asymptomatic breast lump (unknown side)	—	—	99.3	Dense lymphoplasmacytic infiltrates, storiform fibrosis, obliterative phlebitis	47.5	—	—
Pt. 26 (—/—)[31]	Asymptomatic breast lump (unknown side)	—	—	68.3	Dense lymphoplasmacytic infiltrates, storiform fibrosis	45.1	—	—
Pt. 27 (—/—)[31]	Asymptomatic breast lump (unknown side)	—	—	64	Dense lymphoplasmacytic infiltrates, storiform fibrosis, obliterative phlebitis	74.1	—	—
Pt. 28 (—/—)[31]	Asymptomatic breast lump (unknown side)	—	—	52.7	Dense lymphoplasmacytic infiltrates, storiform fibrosis, histiocytes	57	—	—
Pt. 29 (—/—)[31]	Asymptomatic breast lump (unknown side)	—	—	48.7	Dense lymphoplasmacytic infiltrates, storiform fibrosis	59.6	—	—
Pt. 30 (—/—)[31]	Asymptomatic breast lump (unknown side)	—	—	42.3	Dense lymphoplasmacytic infiltrates, storiform fibrosis, obliterative phlebitis, histiocytes	32	—	—
Pt. 31 (—/—)[31]	Asymptomatic breast lump (unknown side)	—	—	41.7	Dense lymphoplasmacytic infiltrates, storiform fibrosis	40.2	—	—

Pt. # (age, y/sex)ref.	Presentation	Imaging	Biopsy	Serum IgG4 (mg/dL)	Histopathology	% $IgG4^+/IgG^+$	Treatment	Outcome
Pt. 32 (—/—)[31]	Asymptomatic breast lump (unknown side)	—	—	41	Dense lymphoplasmacytic infiltrates, storiform fibrosis	40.5	—	—
Pt. 33 (—/—)[31]	Asymptomatic breast lump (unknown side)	—	—	31	Dense lymphoplasmacytic infiltrates, storiform fibrosis, obliterative phlebitis	62	—	—
Pt. 34 (—/—)[31]	Asymptomatic breast lump (unknown side)	—	—	28.7	Dense lymphoplasmacytic infiltrates, storiform fibrosis, obliterative phlebitis, histiocytes	18.9	—	—
Pt. 35 (—/—)[31]	Asymptomatic breast lump (unknown side)	—	—	27.3	Dense lymphoplasmacytic infiltrates, storiform fibrosis, obliterative phlebitis	49.4	—	—
Pt. 36 (—/—)[31]	Asymptomatic breast lump (unknown side)	—	—	26.3	Dense lymphoplasmacytic infiltrates, storiform fibrosis, obliterative phlebitis	61.2	—	—
Pt. 37 (—/—)[31]	Asymptomatic breast lump (unknown side)	—	—	19.7	Dense lymphoplasmacytic infiltrates, storiform fibrosis, obliterative phlebitis	34.1	—	—
Pt. 38 (—/—)[31]	Asymptomatic breast lump (unknown side)	—	—	17.7	Dense lymphoplasmacytic infiltrates, storiform fibrosis, histiocytes	49.5	—	—
Pt. 39 (—/—)[31]	Asymptomatic breast lump (unknown side)	—	—	16.7	Dense lymphoplasmacytic infiltrates, storiform fibrosis, obliterative phlebitis	42	—	—
Pt. 40 (—/—)[31]	Asymptomatic breast lump (unknown side)	—	—	12	Dense lymphoplasmacytic infiltrates, storiform fibrosis, obliterative phlebitis, histiocytes	46.2	—	—
Pt. 41 (82/F)[32]	Asymptomatic right breast lump	**MG:** Ill-defined focal asymmetric infiltrates, no calcifications<br>**US:** Ill-defined heterogeneous mass	Core biopsy	—	Polyclonal T and B cell infiltrates, macrophages, plasma cells, histiocytes, lymphoplasmacytic infiltrates with fibrosis and sclerosis	—	—	—
Pt. 42 (65/F)[33]	Asymptomatic right breast lump	—	Excisional biopsy	150	Robust lymphocytes and plasma cells, diffuse fibrosis, $IgG4^+$ cells 172/HPF, few obliterative phlebitis with concomitant features of Rosai-Dorfman disease	82	Excision	No recurrence

Pt. # (age, y/sex)[ref.]	Presentation	Imaging	Biopsy	Serum IgG4 (mg/dL)	Histopathology	% IgG4+/IgG+	Treatment	Outcome
Pt. 43 (78/F)[33]	Asymptomatic right breast lump	**PET:** Right breast mass and right axillary lymph nodes with high FDG uptake	Excision	18	Robust lymphocytes and plasma cells, diffuse fibrosis, IgG4+ cells 118/HPF, few obliterative phlebitis with concomitant features of Rosai-Dorfman disease	53	Excision	No recurrence
Pt. 44 (54/F)[34]	Asymptomatic right breast lump	**MG:** Irregular-shaped, dense lesion with indistinct margins<br>**US:** Low-echoic lesions<br>**CT:** For eyelids assessment, breast mass noted<br>**MRI:** Segmental enhancement	Core needle biopsy	217	Irregular stromal cell proliferation associated with severe lymphoplasmacytic infiltration, adenosis, and intraductal papilloma surrounded by fibrosis; diffusely distributed IgG4+ plasma cells	—	Prednisone 40 mg/day tapered to 10 mg/day maintenance therapy	No recurrence
Pt. 45 (43/F)[35]	Asymptomatic right breast lump	**US:** Low-echoic breast mass with abundant vascular signal<br>**PET:** Right breast mass and right axillary lymph nodes with high FDG uptake<br>**MRI:** Irregular shaped contrasted mass with enhancement	Extraction	517	Dense fibrosis with abundance of plasma cells	70	Prednisone 30 mg/day tapered to 5 mg/day maintenance therapy	No recurrence
Pt. 46 (62/F)[36]	Asymptomatic right breast lump	**MG:** Nodule, right lower portion<br>**US:** Hyperechoic nodule in right lower portion	Performed for diagnosis and surgical extraction	—	Large polygonal histiocytes with zonal proliferation pattern, lymphoplasmacytic cells with stromal fibrosis with concomitant features of Rosai–Dorfman disease; positive reactions for IgG and IgG4 in fibrotic stroma	56.7	Surgical extraction	No recurrence
Pt. 47 (69/F)[37]	Asymptomatic left breast lump	**MG:** Bilateral diffuse calcifications and multiple circumscribed masses in the medial left breast<br>**US:** Multiple masses in the left breast and chest wall; abnormal left axillary lymph nodes<br>**MRI:** Multiple heterogeneous enhancing masses in left breast and chest wall surrounding and involving multiple ribs	US-guided	191	Mixed inflammatory infiltrate with background fibrosis; no staining for IgG4 was performed	—	—	—

Pt. # (age, y/sex)$^{ref.}$	Presentation	Imaging	Biopsy	Serum IgG4 (mg/dL)	Histopathology	% $IgG4^+/IgG^+$	Treatment	Outcome
Pt. 48 (46/F)[38]	Indurative left breast lump	**MG and US:** Ill-defined nodule<br>**MRI:** Breast mass with enhancement	Excisional	185	Irregular proliferation of stromal cells associated with severe lymphoplasmacytic infiltration, obliterative phlebitis, and eosinophils; many plasma cells within the lesion immunohistochemically positive for IgG4	—	—	—

—, not known; CT, computed tomography; F, female; FDG, fluorodeoxyglucose; HPF, high-power field; M, male; MG, mammography; MRI, magnetic resonance imaging; MTX, methotrexate; PC, plasma cells; PET, positron emission tomography; US, ultrasonography.
